# Development of a physical literacy consensus statement for Chile: study protocol

**DOI:** 10.3389/fpubh.2025.1554070

**Published:** 2025-05-14

**Authors:** Jaime Carcamo-Oyarzun, Catalina Rivera-Gutierrez, Luis Henriquez-Alvear, Pedro Delgado-Floody, Cristina Ferbol, Mauricio Diaz-Alvarado, Pablo Cumilef-Bustamante, Nicolas Martinez-Lopez, Paula Guarda-Saavedra, Paulina Candia-Cabrera, Gustavo Pavez-Adasme, Marcelo Castillo-Retamal, Rodrigo Vargas-Vitoria, Jessica Ibarra-Mora, Luis Veas-Alfaro, Rodrigo Diaz-Guaita, Luis Añazco-Martinez, Isaac Estevan

**Affiliations:** ^1^CIAM Physical Literacy Research Centre, Faculty of Education, Social Science, and Humanities, Universidad de La Frontera, Temuco, Chile; ^2^Department of Physical Education, Universidad de La Frontera, Temuco, Chile; ^3^Escuela de Posgrado, Vicerrectoría de Investigación y Posgrado, Universidad Católica de Temuco, Temuco, Chile; ^4^Departamento de Educación y Humanidades, Universidad de Magallanes, Punta Arenas, Chile; ^5^Instituto Nacional de Deportes Región de Aysén, Coyhaique, Chile; ^6^Grupo de Investigación AFSYE, Universidad Adventista de Chile, Chillán, Chile; ^7^Departamento de Ciencias de la Actividad Física, Universidad Católica del Maule, Talca, Chile; ^8^Departamento de Educación Física, Deportes y Recreación, Universidad Metropolitana de Ciencias de la Educación, Santiago, Chile; ^9^Facultad de Educación, Universidad Central Sede Coquimbo, La Serena, Chile; ^10^Departamento de Educación Física, Deportes y Recreación, Universidad de Atacama, Copiapó, Chile; ^11^Departamento de Educación Física, Universidad Arturo Prat, Iquique, Chile; ^12^Activitat Física i Promoció de la Salut (AFIPS) Research Group, Department of Teaching of Physical Education, Arts, and Music, University of Valencia, Valencia, Spain

**Keywords:** physical literacy, physical education, physical activity, public policies, consensus

## Abstract

Physical literacy is a multidimensional concept that encompasses various aspects of an individual’s ability to engage in physical activities throughout their lifespan. As a holistic approach, physical literacy (PL) has generated a lot of interest in the field of physical education, physical activity, and health promotion. Despite its growing popularity, the conceptualization and understanding differ among the countries. In the case of Latin America, the concept of physical literacy is still relatively new and influenced different countries, making complex the contextualization for this region. Considering some aspects of public policies that seek to promote physical activity in Chile, such as the national policy of physical activity and sport or the curricular bases of the subject of physical education, this paper seeks to propose the systematized elaboration based on evidence to generate a contextualized consensus with the different actors that can contribute to the development of physical literacy in Chile. This protocol will involve mixed methods throughout the implementation of six work package processes: (1) review of the existing evidence about physical literacy for its understanding in Spanish-speaking populations, (2) analysis of drawings (preschoolers), semi-structured interviews (children, adolescents, and adults), and focus groups (older adults) to investigate the community’s view of physical activity, (3) discussion groups and nationwide online questionnaire to analyze the understanding of the concept of PL by both physical education teachers and political actors, (4) discussion groups to consolidate community perceptions on the relevance of physical activity and physical literacy to prepare a preliminary consensus, (5) national consultation on the draft Physical Literacy Consensus Statement text, and (6) discussion and definition of the Physical Literacy Consensus Statement for Chile. This consensus will help stakeholders, teachers, researchers, and the community in general to understand a common concept of physical literacy, which will allow its development, promotion, and application in the Chilean context. Likewise, this work could be a spearhead to the implementation of physical literacy in Latin America and low-and middle-income countries in other regions.

**Clinical trial registration:**

The clinical trial will be registered on ClinicalTrials.gov under the name CONALMOT_CL (COnsenso Nacional en ALfabetizaic MOTriz in Spanish: Physical Literacy National Consensus).

## 1 Introduction

The benefits of regular physical activity (PA) throughout life are well documented, and its contribution to people’s physical and mental health and general wellbeing has been recognized (1). For children and adolescents, the contribution of PA becomes even more relevant, as there is evidence to demonstrate multiple benefits for their fundamental development, enhancing physical, psychological, cognitive, and social aspects (2–4). Nevertheless, there is a high prevalence of physical inactivity in the pediatric population worldwide, which has become a major concern for our society (5). It is estimated that 80% of children and adolescents do not meet the minimum recommendations of 60 min per day of moderate and vigorous intensity PA (5). This problem has become even more dramatic in Chile, with only 3 out of 10 children meeting international PA recommendations (6, 7). In the Global Matrix 4.0 on Physical Activity for Children and Youth, which provides the results from the Report Card on Physical Activity for Children and Youth, Chile ranks second-to-last in overall PA (5). Post-pandemic studies have shown that this situation has worsened, with an increase in sedentary behaviors (8), low levels of PA (9) and motor competence (10). Despite the efforts made to reverse this situation, with attempts to implement PA programs and promote children’s sports (11, 12), it has not been possible to reverse the trend of high levels of physical inactivity reported in recent years (5, 13, 14). Given the situation, measures must be taken to counteract this problem, which should not only focus on temporary programs offering PA but also urgently incorporate comprehensive approaches that encourage the appreciation of and commitment to perform PA regularly throughout life.

A comprehensive approach that has emerged as an alternative for the development of regular PA throughout life is physical literacy (PL) (15), which is understood as the motivation, confidence, physical competence, knowledge, and understanding to value and engage in lifelong physical activity (16). As a holistic approach, PL is seen as a significant human capability that can be developed by anyone (16), which has a philosophical argumentation related to monism, existentialism, and phenomenology (17) and which bases its integral perspective beyond physical health, emphasizing the dimension of corporeality and the intrinsic value of the practice of lifelong PA (16). While this PA-related concept is not new (18), in recent years, this approach has garnered great interest among the scientific community and has positioned itself as one of the emerging and most popular topics in the field of physical education (PE), health and sports, being regarded as one of the main foundations for promoting high-quality physical education (19) and fostering healthy lifestyles (20–22). Due to its holistic perspective, PL adopts a very attractive approach to managing PA in public policy, so its use has been addressed not only by researchers but also by political decision makers (23). Consequently, the dissemination of the term PL has reached different countries; however, the conceptualization and structure of this approach are understood differently depending on both geographical location and context (23, 24). In the case of Chile, the use of the PL concept is in the early stages. A narrative review of Chilean physical activity public policies considers PL only as an approach for the promotion of physical activity in childhood and not for adulthood (25). There is also evidence that knowledge of the concept of PL among Chilean physical education teachers is limited, with a predominant focus on the physical aspects (26). Even a new Physical Education curriculum proposal for Chile proposes PL as a grouping of objectives about physical fitness and training, disregarding the psychological, social and cognitive dimensions of PL (27). This scant evidence makes it clear that all the actors working in the field of PA need to agree on what is understood by PL and on the structure that will enable its implementation. To this end, it is necessary to analyze Chile’s public sports and physical education policies, which can be strengthened with the PL approach.

In Chile, the Ministry of Sports is the governmental institution responsible for developing sports and PA policies (28). Its main objective is to propose and evaluate the National Physical Activity and Sports Policy and general plans in the field of sports (28). However, it does not directly execute actions related to the national PA and sports policy because this executive action is the responsibility of the National Institute of Sport (29). This has representation in each of the regions in Chile to reach the entire population and apply the programs emanating from the National Policy (29). The current National Physical Activity and Sport Policy, in force since 2016, is the management instrument for plans, programs, and actors (public and private) to promote social integration through PA and sports (11). Its purpose is to promote the integral, individual, and community development of the population through the systematic practice of PA and sports throughout life, from a legal approach that safeguards gender equity, interculturalism, and social inclusion in its broadest sense (11). In order to update this National Policy, a public consultation has been called to build the new National Physical Activity and Sports Policy 2026–2037 (30). This offers an opportunity for PL to be considered an approach to aid in determining this policy framework since, despite the positive assessment of the National policy as a document, applying high-standard policies to PA indicators requires additional or more complex intermediate strategies. However, there is a need for a common concept of Physical Literacy, which can be understood by all those involved in implementing these public policies.

In addition to promoting sports policies, the Ministry of Sports coordinates a National System of Physical Activity and Sport (11). Its purpose is to coordinate the actions of public institutions (Ministry of Health, Ministry of Education, Ministry of Housing and Urbanism, Ministry of Labor, Ministry of Social Development, among others) and private institutions (sports clubs, companies, universities, among others). Although the national strategy does not specify how these institutions are organized, some programs perform intersectoral work to promote PA and sports. This is the case of the public program “Elige Vivir Sano” (“Choose to Live Healthy”), created in 2010 and transformed into law in 2013 (31), the aim of which is to promote healthy lifestyles to improve people’s quality of life and wellbeing. This program is a management model consisting of policies, plans, and programs developed and implemented by different government agencies aimed at contributing to the generation of healthy lifestyles and the prevention and reduction of risk factors and behaviors associated with noncommunicable diseases (31). It comprises an interministerial committee with the participation of 10 government ministries, which make available some of their programs to promote PA among the population and thus promote healthy lifestyles (32). Although this system seeks to combine the efforts of different ministries to optimize outcomes, the problems that the “Elige Vivir Sano” program endeavors to solve are sedentary lifestyles and reducing the risks of the population acquiring a chronic noncommunicable disease, which gives a functionalist meaning to the promotion of sports and health care (33), sliding back into the dualistic and salutogenic viewpoint (33, 34) that Chilean public policies give PA, without considering the all-encompassing view needed to achieve lasting effects. Notwithstanding, PL integrates such all-encompassing health views, which might involve a holistic and appropriate approach to new National Strategic Plans for Physical Activity and Sports.

Another important aspect for the development of PL as a public policy has to do with the Physical Education curriculum. PL is embodied in PE (35), and its development has been considered one of the main goals of PE (19), so it is necessary to understand how PL can relate to its curriculum. In Chile, the school subject is called “Physical Education and Health” (PEH), and as this name reflects, it is health-centered (36), without evidence to indicate an approach to PL as such. However, some programs seek to improve the quality of life through the practice of PA to curb the levels of obesity and chronic non-communicable diseases (25). The curriculum emphasizes developing PA habits, indicating that the subject is oriented toward making the habits of an active and healthy life and the regular practice of PA a central part of young people’s lives, both in and out of school (37). The genesis of the PEH curricular foundations is directly linked to the poor outcomes of a national physical fitness assessment (38); this was directly related to the quality of PE classes, misunderstanding the overall purpose of this subject, and focusing on the development of a physical condition that seeks to improve schoolchildren’s physical health (39). Despite this biomedical emphasis, the curriculum states that PEH is a central subject of school education, which is part of the integral formation process of the human being (37, 40). Although this statement of comprehensiveness is repeated in other sections of the document, particularly in the attitudinal objectives or disciplinary knowledge, its application is not reflected in the learning outcomes (41). Hence, the PE teacher fulfills the fundamental role of curriculum implementation for schoolchildren’s learning (42). Their beliefs may influence the application of the curriculum in their classes, reproducing the traditional perspective linked to health, fitness, and sports or applying a holistic view of PE that seeks to develop not only the physical but also the emotional and the understanding and meaning of physical activity (43). Although it is recognized that PE should be directed toward the integral development of the person, there are still practices in which physical and cognitive aspects are prioritized (26).

With the intention of updating the curriculum and giving it a more comprehensive look, the Chilean Ministry of Education presented a Curriculum Updating Proposal for all subjects, including Physical Education and Health (27). In the preliminary proposal, the Ministry of Education considers PL as an axis of objectives. However, these objectives only deal with aspects linked to physical training, without considering other dimensions of human development. This reduction of PL can generate misunderstandings among teachers, so it is necessary that the new curriculum, which will be valid in 2026, presents a clear conceptualization of Physical Literacy and includes the development of psychological, social and cognitive aspects (44). By offering a clear concept of PL, which is understood equally by the teachers, it might be possible to collaborate in fulfilling the learning objectives stipulated in the curriculum from a holistic perspective (19).

This succinct review of some of the public policies to promote PA in Chile shows a deep-rooted biomedical perspective centered on the physical aspect of bodily development (33, 34); however, these various public policies seek a person’s comprehensive development (11, 31, 37). This apparent dissonance between a search for comprehensive development based on the proposal for actions concerned only with the physical sphere makes it necessary to consider a holistic approach that also involves psychological, cognitive, and social aspects. PL offers an alternative that can contribute to a person’s comprehensive development through PA (45), providing a clearer framework for reconceptualizing and reorganizing strategic policies to promote education, sports, and health (46). However, this approach and its structure cannot be translated literally from English to Spanish without considering the country’s cultural aspects, so a discussion of its relevance to the Chilean context is needed. Although scientific evidence highlights the need for a definition adapted to each culture and context (47, 48), there is scarce literature that addresses the concept of PL in a contextualized manner in Spanish-speaking regions. Thus, for PL to truly contribute to the promotion of PA in Chile, the concept and its structure must be contextualized to the country’s reality, considering the community’s views on the relevance and commitment to lifelong PA. Therefore, this manuscript proposes a study protocol to determine a consensus statement on the concept and structure of PL contextualized for Chile.

## 2 Materials and methods

To generate a consensus on PL for Chile, a mixed study design that combines qualitative and quantitative methodologies will be implemented in six work packages (WP). Being a national study, it is expected that people (e.g., PE and health professionals, stakeholders, etc.) from all regions of Chile will participate in the different stages of the research. Depending on the WP, participants will be organized by age range, from preschoolers to older adults. A working group dedicated to indigenous peoples will also be considered, in order to understand the indigenous communities’ vision of their embodied practices. Similarly, efforts will be made to enlist relevant actors promoting PA and fulfilling professional or organizational roles in education, sports, and health.

### 2.1 Work packages

#### 2.1.1 Package 1

The objective of this WP1 will be to conduct a systematic review to analyze the different definitions and domains that comprise PL in the literature. The PRISMA (Preferred Reporting Items for Systematic Reviews and Meta-Analyses) (49) will be used to perform a bibliographic search on four platforms, including digital databases: EBSCOhost (through CINAHL Complete, ERIC and SPORTDiscus), ProQuest (through APA Psycinfo, Education Collection, and ProQuest Central), Web of Science (through MEDLINE, SciELO citation index and Web of Science Core Collection) and Scopus. The search strategy will be carried out considering the title, abstract, and keywords by adopting the following query string: [“Physical litera*” AND (“defin*” OR “model” OR “concept” OR “concept*” OR “theor*” OR “structur*”)]. This review has already been registered in INPLASY with the registration code 202230074 (50).

#### 2.1.2 Package 2

In the WP2 the aim is to investigate the community’s view of PA and their willingness to practice it regularly. For this purpose, different data collection strategies will be used, initially using a qualitative perspective. The choice of a qualitative study is based on the possibility of collecting data with a high component of meanings and diversity starting from inductive logic and a nomothetic approach (51). The application of these methodologies will be structured based on the age ranges of the people who make up the community, organized as follows:

##### 2.1.2.1 Preschoolers: analysis of drawings

The participation process of preschool children in this study will be developed through drawings, where they will be asked to exhibit their representations related to PA. The use of drawings is due to the young age of the participants, considering that expressing ideas through images can offer greater access and understanding of what they wish to communicate (52). Similarly, the meaning and explanation of the drawings will be accompanied by a conversational process, and it is anticipated that the children will comment and express their representation through both explicit and implicit discourse (52).

##### 2.1.2.2 Children, adolescents, and adults: semi-structured interviews

An interview technique will be used to learn about the views of children, adolescents, and adults. To this end, the research is founded on recognizing children and adolescents as people with rights, where their voice acquires relevance, understanding that their participation contributes to the development of their life trajectories (53). The interview should consider the analysis of the meanings expressed “between the lines” and at the factual level, which will make it possible to know and recognize the world of the respondent’s life through their interpretation (54). In this sense, two essential processes will be carried out before proceeding with the formal interviews: (a) explaining in detail and notifying in advance the topics to be discussed in the interview, and (b) conducting a pilot test of the interview. These actions are necessary to ensure compliance with the objectives, relevance, timing, and responses (55). Specifically, a semi-structured interview is proposed, as this provides access to the implicit theories of the subjects, rendering their knowledge about a topic explicit in a simultaneously organized and freeway (56). The interviews will be recorded through memos and audio recordings to create transcripts that provide the greatest accuracy of the narratives and thus achieve an interpretive analysis that adheres to the intention of what was said by the respondents (55, 57). The information collected will be processed using qualitative methods, specifically grounded theory. For this purpose, the ATLAS.ti (Version 23) software will be used.

##### 2.1.2.3 Older adults: focus groups

The work undertaken with older adults will be group work, taking advantage of the opportunity provided by the various structured and recognized organizations of older adults in Chile, which bring together retirees over 60 years of age (58). These groups are usually self-managed or linked to health centers and provide care services and leisure and recreational activities. Therefore, the application of focus groups in this group is highly relevant, understanding that this technique allows people to develop consensus and ideas naturally and with less chance of being intimidated during the data collection process since the participants are in a familiar environment with people they know (57). The data collected will be analyzed based on Grounded Theory (59), which will make it possible to propose inferences and advance interpretations related to the views of PA held by this age group.

##### 2.1.2.4 Indigenous peoples: semi-structured interviews

Chile has different native peoples, whose indigenous ethnic groups are geographically spread all over Chile, from north to south. The Indigenous Peoples of Chile have diverse ancestral practices that involve, among others, ceremonies and traditional games that include multiple physical expressions, and that count on the active participation and collaboration of the entire community (60). For this reason, it is relevant to consider the vision that Indigenous Peoples have about their physical identity and physical activities which in turn might impact the consensus statement on the concept and structure of PL in Chile. As with the data collection corresponding to the group of children and adults, we will use the semi-structured interview technique in order to ascertain the vision of people with Indigenous backgrounds. Considering the characteristics of each Region, the researchers will be guided by all the cultural protocols of approach and conversation with the indigenous communities (61). To this end, we will carry out a formal application process for the project in each of the communities, through their leaders and traditional agents (62). This part of the study will be carried out by researchers with previous links to indigenous peoples and experience in intercultural analysis, to ensure respect for the culture and to give confidence to the interviewees. The methods of analysis we will use for the analysis of the data collected will be based on Grounded Theory (59).

#### 2.1.3 Package 3

Parallel to WP2, WP3 aims to analyze the understanding of the concept of PL by both physical education teachers and political actors. Qualitative and quantitative methodologies will be used in this package, organized as follows:

##### 2.1.3.1 Discussion groups

The discussion group is expected to be applied in different regions of the country to collect information that will address all areas of Chile to obtain content enriched by the country’s social, cultural, and geographic diversity. Each discussion group will be composed of three types of relevant actors: (a) decision makers from the fields of education, sports, and health, (b) physical education teachers working in schools and PA promotion programs, and (c) coaches and trainers working in government sports programs. Discussion groups are a qualitative technique, which is assumed to be different from group interviews or focus groups, as they can work from the discussion on a particular topic mediated by the research team and not the application of a list of questions in front of a group (63). Discussion group research has been widely used in various fields of knowledge, including the social sciences and education, and is a relevant strategy for this study. Likewise, discussion groups are a technique that generates meaning and not only information, i.e., a conversation that seeks to form meanings among the participants (64). Two elements must be considered for the proper development of discussion groups: first, a thematic guide must be created to encourage and guide the discussion; second, the correct composition of the group, because common points must be ensured among the participants to achieve an appropriate debate that allows for consensuses and differences. It is important to consider the experience and knowledge of the participants (63).

##### 2.1.3.2 Nationwide online questionnaire

This questionnaire will compile PE teachers’ current understanding of PL in the different regions of Chile. Its use seeks to ask about the knowledge of PL on a national level. The decision to use this instrument is based on the potential for large-scale data collection, with standardization being one of the theoretical foundations to be considered to expose each participant uniformly to the response production process (65).

#### 2.1.4 Package 4

The WP4 is meant to consolidate community perceptions on the relevance of PA and PL to prepare a preliminary consensus. WP4 will involve academic experts from various disciplines (education, health, psychology, philosophy, among others), who will collaborate with their experience and knowledge to consolidate the perceptions of the community and decision makers to develop a common understanding of PL that will generate the basis for a preliminary conceptualization. For this, the discussion group technique will be used again. Discussion groups seek to reach a consensus through group interaction, i.e., the ideas that arise in the interaction of the discussion group present questions, disagreements, and nuances that make it possible to reach a consensus on a particular topic (65). Based on the information gathered from the previous WPs, the expert academics will debate the categories emerging from the views of the community, decision makers, and PE teachers to propose a consensus that can harmonize a conceptualization of PL. Unlike the WP3 discussion groups, the research questions will be much more specific in this WP, focusing on the relevant aspects from the previous WPs. Thus, the use of this discussion group takes on a pragmatic purpose (56), which in this case is the preliminary proposal of the consensus.

#### 2.1.5 Package 5

The WP5 purpose is to receive feedback from experts in the sectors of sport, PA, health, education, the community, and decision makers and collect their opinions on the preliminary proposal of the Physical Literacy Consensus for Chile resulting from the discussions of the expert group in WP4. To this end, and to encourage the participation of all stakeholders interested in promoting PA, a National Consultation will be conducted online, which will present a draft consensus, where a preliminary definition of PL according to the different actors of the Chilean community will be outlined, hoping to receive feedback to optimize this preliminary consensus.

#### 2.1.6 Package 6

**Figure 1:**
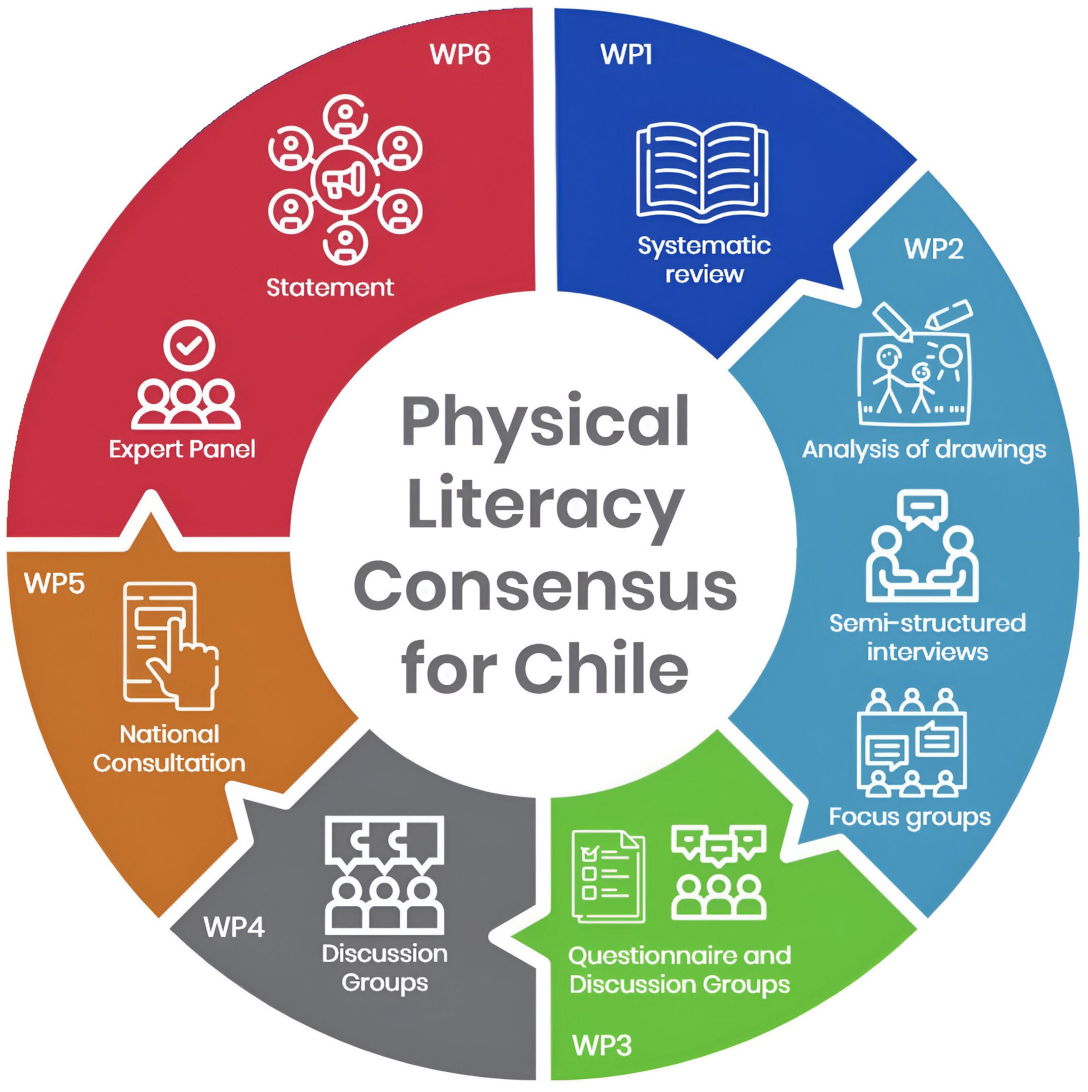
Work packages for the physical literacy consensus study in Chile.

Finally, based on the National Consultation, WP6 aims to prepare a statement on the concept and structure of PL suitable to our context and the argumentation of why it is important for the Chilean community. This process will be undertaken through discussion groups with academics, understanding that the experts are symmetrical in terms of their opportunities for participating, discussing, arguing, and explaining their points of view (63) (Figure 1).

### 2.2 Logistical procedures

The various WPs mentioned above involve a wide deployment within a country with a large geographic area. To this end, each WP will be coordinated by academics from the Physical Literacy Research Center [(Centro de Investigación en Alfabetización Motriz (CIAM) in Spanish)] at the Universidad de La Frontera. Likewise, the CIAM has associated researchers from other universities and other regions of Chile, encompassing the entire nation, who will coordinate the application of the WPs in their respective regions.

### 2.3 Ethical aspects

The study will be submitted for review by the Scientific Ethics Committee of la Universidad de La Frontera. Among the ethical considerations, authorization will be requested from parents or guardians for the participation of minors, and they will have to sign an informed consent form. Additionally, minors will consent to participate, while those of legal age shall give their consent in writing. The ethical standards and principles of the Declaration of Helsinki for research involving human subjects will be always respected. This study will be characterized by voluntary participation and freedom to withdraw from the study at any time should any participant wish to do so.

## 3 Expected outcomes and discussion

This manuscript proposes a protocol to determine a consensus on the concept and structure of PL contextualized to the Chilean reality. This study uses a mixed method approach to gather opinions from the Chilean community on the importance of PA so that, when combined with the ideas that teachers and decision makers have about PL, a consensus can be reached on what is understood by PL in Chile. It is expected that this study will contribute to harmonizing criteria for applying strategies aimed at developing PL. In addition, it is likely to contribute to aligning certain Chilean public policies related to education, sports, and health, which, although their goal is people’s comprehensive development, are mainly focused on the physical dimension (33, 34). Having all the actors involved in the promotion of PA share the same understanding of the concept of PL and agree on the need to work on the same dimensions will give the intentions outlined in the theory a greater impact on their implementation (48).

Each WP corresponds to a block for creating a shared understanding of a conceptualization of PL. In the first WP, it is fitting to begin this project with a systematic review of the concept of PL to understand it from a global context. Despite the worldwide interest in PL, its conceptualization remains elusive due largely to different theoretical perspectives and contexts (66). Although several systematic reviews have been performed on the conceptualization and structure of PL (66–69), to date, there has been no systematic review in Spanish oriented to Latin American culture. Therefore, the product of this package will not only help build a consensus in Chile but will also be a pioneering contribution to the contextualized dissemination of PL in Spanish-speaking countries.

For the second WP, focused on the community’s views on the importance of PA, all age ranges are to be represented to understand the relevance of corporeality as a lifelong human skill. In the case of older adults, the role of PA in promoting health and physical independence is crucial, considering that Chile has the highest life expectancy in Latin America (70), but 48.7% of older adults have a low level of PA (71). Knowing older adults’ perceptions will allow us to understand what type of activities they consider appropriate for PA, as well as what factors motivate their participation and commitment to PA programs (72). Similarly, adults’ perceptions can also provide valuable information to understand what they value about PA and what factors could enhance adherence to regular practice (73), especially when 81.3% of the Chilean population over 18 years of age is physically inactive (74). To avoid having only an adult-centric view, it is also relevant to consider the perceptions of preschoolers and schoolchildren, particularly during the formative years when the foundations for a person’s physical literacy are being built and when a solid foundation for PL development throughout life is being laid (75). In addition, this information will be very valuable in linking PL with PE since, in these classes, a variety of experiences can foster or reduce the engagement and enjoyment of bodily practices (76). Regardless of students’ motor performance, there is a motivational disposition toward PE classes, where students who enjoy the activities and feel competent in them undertake more PA (77), so it is important to consider other dimensions beyond the eminently physical. Furthermore, the presence of Indigenous Peoples in this study will strengthen this consensus and offer a contextualization of the culture. In an innovative way, the ancestral perspective of the indigenous communities will be incorporated, which is only present in the PL approach of New Zealand being the only country that considers the spiritual dimension in the consensus (78). All the data collected will provide a wide range of perceptions on the importance of PA throughout life, which will serve as a guiding axis for the conceptualization of PL in the Chilean community.

WP3 involves the relevant actors for the implementation of PL, both in PE, PA programs, and public policies. The participation of PE teachers is crucial, as they are the ones who must promote the development of PL in their classes. In the case of Chile, where the PEH curriculum is characterized by a predominance of biophysical development (34), it is very important to find out what teachers know about PL and their willingness to orient their classes based on this approach. PE teachers have very good acceptance and credibility among schoolchildren (42), so their way of understanding PE will likely be extended to their students. In the case of Chilean teachers, there is a dichotomy in terms of their beliefs regarding their subject, as some teachers reproduce a traditional perspective linked to the development of physical fitness, health, and sport, while others have a more holistic view of PE, seeking overall development (26, 43). Hence, it is quite possible that the intention of incorporating the PL approach as a contribution to Chilean PE may meet with resistance from teachers who adopt a classical perspective or, in contrast, may be favored by teachers who see PE as a means for the comprehensive development of children and adolescents.

Other relevant actors for the PL implementation process are decision makers since their perceptions and beliefs will influence the generation of strategies and public policies to promote PA. Considering that policy development depends on the government of the day and that often a short-term impact is sought to overcome accountability, it is foreseeable that the promotion of PL as a new basic concept for public policies will raise concerns (46); therefore, it is necessary to provide clarity in understanding the concept of PL. Moreover, PL is an attractive concept for producing policies to promote PA, being used in very different ways depending on the stakeholder groups, from combating non-communicable diseases to nurturing the potential embodied in individuals (79). It is therefore important to harmonize criteria so political strategies align and can have a meaningful impact on the community.

The amount of data generated in the previous packages will be analyzed in WP4, where the group of academic experts from various disciplines (health, education, psychology, philosophy, among others) will collaborate with their experience and knowledge to harmonize the perceptions of the community and decision makers to develop a common understanding of PL that will provide the basis for a preliminary conceptualization. Thus, in WP5, the community will be consulted about these preliminary conceptions to receive feedback and confirm the definition and structure of PL that will be understood in Chile. These two WPs (WP4 and WP5) will provide definitive information to solidify the concept of PL in Chile, with WP6 being the epilogue of this process, reviewing the results from the previous WPs and closing with the consensus statement for the development of PL in Chile.

This study is based on a participatory process that will involve many people in various WPs and will not be without limitations. The geography of Chile requires a wide deployment to address all regions, which will imply a high dedication of staff, time, and funds to cover travel. The participation of people not related to physical activity can also be complex, as only people who have or have had some link with physical activity may be interested, so much attention must be paid to the selection of people who will participate in the interviews or focus groups. We recognize the complexities of conducting a study of this magnitude and are aware that the study’s outcomes can be ambitious. Nevertheless, this study has several strengths that will make its implementation feasible, the most relevant of which is its large and important research team. Having a group of academics from different universities in the country, located in different regions, will make it possible to cover the realities of each area of the country and offer greater robustness to each of the WPs, creating a synergy that will strengthen the entire process. Considering that the topic is of great interest for public policies and that some institutions, such as the Ministries of Education, of Sports and of Health have expressed interest in its development, it may be possible to count on support to cover the human and financial needs for its development.

This study will implement methodologies that have, to date, been scarcely used to create public policies that promote people’s comprehensive development through PA. It is expected that their results can generate an alignment in the understanding of the concept of PL, which will facilitate policy decision-making in our context. It will also provide a framework of understanding among PE teachers, who will be able to use the PL approach in their teaching practices, broadening their view beyond the physical realm and reflecting on the importance of the psychological, cognitive, and social dimensions that body practice offers.

We are aware that the PL approach is not a silver bullet that will end physical inactivity and its consequences and that there are many criticisms and doubts about the transfer of theory into practice (80, 81). It is necessary to deepen its study and generate more knowledge about its real impact on the value of PA and a person’s willingness to adhere to its practice throughout life. Precisely for this reason, this study will be a relevant contribution to the study of PL, not only because it will highlight the opportunities and obstacles encountered by the community to value and commit to regular PA but also because it will be a pioneering study in a region that is culturally very different from the countries where PL is currently developed. Therefore, this consensus will not only offer a conceptualization for Chile but also for other Spanish-speaking countries to be guided by this process and its outcomes, enabling them to address the PL approach in a participatory way and focus on the comprehensive development of the Latin American community.
